# Mental Health in Fertile and Infertile Women During the COVID-19 Pandemic: A Comparative Cohort Study

**DOI:** 10.5935/1518-0557.20260035

**Published:** 2026

**Authors:** Pânila Longhi Lorenzzoni, João Paolo Bilibio, Arivaldo José Conceição Meireles, Lígia Konig, Maria Eduarda Branco, João Sabino Cunha-Filho

**Affiliations:** 1 Programa de Pós-Graduação em Ciências Médicas (PPGCM), Universidade Federal do Rio Grande do Sul (UFRGS), Porto Alegre, RS, Brazil; 2 Centro de Reprodução Assistida Fertibc, Balneário Camboriú, SC, Brazil; 3 PRONATUS, Belém, PA, Brazil; 4 Centro Universitário de Brusque (UNIFEBE), Brusque, SC, Brazil

**Keywords:** in vitro fertilization, embryo transfer, infertility, psychological stress, depression, COVID-19 pandemic

## Abstract

**Objective:**

To assess the impact of the COVID-19 pandemic on symptoms of anxiety, depression, and stress among infertile and fertile women.

**Methods:**

This prospective cohort study evaluated psychological distress using the 42-item Depression, Anxiety and Stress Scale (DASS-42) at two time points: during the early pandemic (Phase 1) and after six months (Phase 2). The study included infertile women whose embryo transfers were postponed (n=71) and fertile women who had given birth over six months earlier without fertility issues (n=117). Ethical approval was obtained (CAAE: 32964620.3.1001.5334).

**Results:**

The mean age was higher in the infertile group (37.8 *vs*. 35.5 years; *p*=0.001). In Phase 1, infertile women reported significantly higher levels of stress (7.7 *vs*. 5.6; *p*=0.018) and depression (4.0 *vs*. 2.3; *p*=0.002), with no significant difference in anxiety (3.4 *vs*. 2.7; *p*=0.389). In Phase 2, stress (3.7 *vs*. 3.1; *p*=0.201), depression (4.0 *vs*. 3.5; *p*=0.446), and anxiety (5.8 *vs*. 5.4; *p*=0.666) scores were similar between groups. Longitudinal analysis in both infertile and fertile groups showed a significant reduction in stress and an increase in anxiety. Depressive symptoms worsened in the fertile group (*p*=0.018).

**Conclusion:**

Infertile women initially showed higher stress and depression than fertile controls. Over six months, stress declined and anxiety increased in both groups; depressive symptoms remained stable in infertile women but slightly worsened among fertile women. These findings support the inclusion of targeted and ongoing mental health support as part of fertility care.

## INTRODUCTION

The pandemic caused by the SARS-CoV-2 virus introduced significant challenges worldwide, where the emergence of a highly infectious and unprecedented disease generated widespread uncertainty (Hao *et al*., 2022). During this period, elective medical treatments, including routine surgeries and non-urgent procedures, were suspended in many regions as health systems redirected resources toward COVID-19 care (Tippett, 2022). In Brazil, most assisted reproductive treatments (ART), such as in vitro fertilization (IVF), intrauterine insemination (IUI), and ovulation induction, were paused following guidance from fertility associations like SBRA and REDLARA during the height of the health crisis (Souza *et al*., 2020).

Delays in treating infertile couples, who are already vulnerable to elevated levels of anxiety, stress, and depression, may exacerbate these psychological burdens (Massarotti *et al*., 2019). Infertility is defined as the failure to achieve pregnancy after 12 months of regular unprotected intercourse and may persist for several years in many couples. Emotional suffering in these individuals is frequently characterized by frustration, loss of control, and clinically relevant symptoms of anxiety and depression; meta-analyses estimate pooled prevalences of ~36% for anxiety and ~28-44% for depression among infertile women (Kiani *et al*., 2020; 2021). During the COVID-19 pandemic, professional societies recommended pausing assisted reproduction, and many programs suspended new treatment cycles-changes perceived by patients as highly distressing (Monteleone *et al*., 2020, Sauer-Zavala *et al*., 2021).

The prolonged duration of infertility treatment has consistently been associated with psychological symptoms. The additional delays caused by the pandemic may have further increased anxiety and emotional distress (Lawson *et al*., 2021). In this context, ART suspensions contributed to greater emotional vulnerability among patients. Women whose treatments were delayed reported increased anxiety, distress, and a greater sense of anguish including intensified depressive symptoms (Tippett, 2022).

Infertility treatment is described by many couples as one of the most stressful events of their lives. Even amid the uncertainties of COVID-19, infertility itself remained a primary emotional stressor. Additionally, infertility remains perceived as a top stressor for patients-often matching or exceeding the stress attributable to the pandemic itself (Vaughan *et al*., 2020).

However, only a limited number of longitudinal studies have compared infertile and fertile women to evaluate how psychological distress evolves over time in response to major external stressors (Bagade *et al*., 2022, Vahratian *et al*., 2011). We proposed that infertile patients may present distinct baseline levels of stress, anxiety, and depression compared with fertile controls, and that their trajectories of these symptoms following a crisis may differ. Therefore, this longitudinal, comparative investigation aims to fill that gap by evaluating and contrasting symptom evolution in both groups.

Knowing that infertility itself is a very stressful event for couples, increasing stress, anxiety, and depression, and that the COVID-19 pandemic may have worsened these symptoms, comparing how these symptoms evolved compared to fertile couples would be important to understand and be able to help infertile couples on their journey to assisted reproduction. Therefore, the objective of this study was to analyze the impact of the COVID-19 pandemic on the levels of anxiety, depression, and stress in infertile and fertile women.

## MATERIAL METHODS

### Study Design and Ethics

This prospective cohort study was conducted with infertile and fertile women at the onset of the COVID-19 pandemic. Eligible participants were recruited from a reproductive medicine reference center and selected based on specific inclusion and exclusion criteria. Data collection occurred between July 2020 and July 2021. All participants received oral and written instructions regarding confidentiality and provided informed consent prior to participation.

The study was approved by the National Research Ethics Committee and by the Ethics Committee of the Institute of Health Sciences, Federal University of Pará (CAAE: 32964620.3.1001.5334). The protocol also received review and endorsement from the Research Ethics Committee (CEP) of the Federal University of Rio Grande do Sul (UFRGS). All procedures followed the guidelines outlined in Brazilian research regulations (CNS Resolution 466/12 and 510/2016).

### Participants

**Figure 1:**
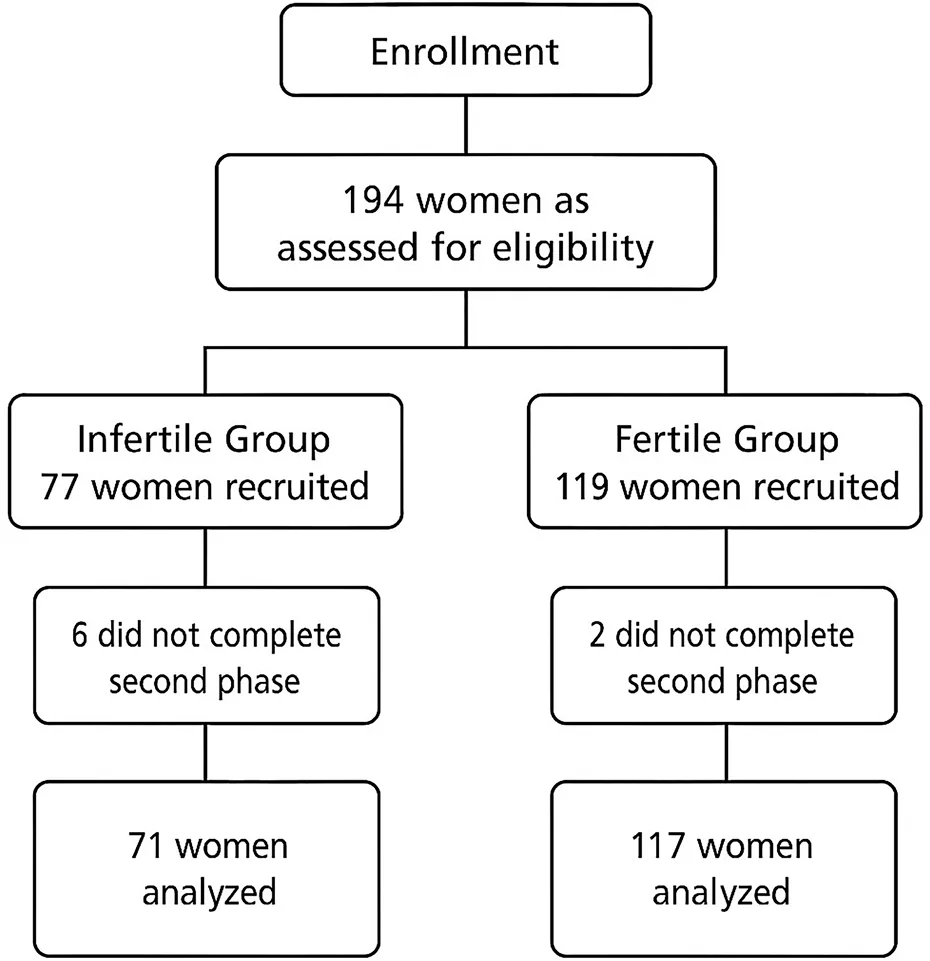
CONSORT flow diagram illustrating the recruitment, exclusion, and final inclusion of infertile (n=71) and fertile (n=117) women evaluated in both study phases

A total of 196 women were initially recruited, including 77 infertile patients scheduled for in vitro fertilization (IVF) and 119 fertile women who had delivered a child more than six months before the study. After follow-up, 71 infertile and 117 fertile participants completed both study phases and were included in the final analysis. Six infertile and two fertile participants were excluded due to loss to follow-up or incomplete responses in the second phase (Figure 1).

Infertile group: Women with a clinical diagnosis of infertility for over one year, scheduled for in vitro fertilization (IVF), and whose embryo transfers were postponed due to the COVID-19 pandemic.

Fertile group: Women who had delivered a child more than six months before the study and reported no history of infertility.

### Procedures - Phase One

During the first phase, conducted between July and December 2020, during the early stage of the COVID-19 pandemic, participants were initially invited to complete an anonymous, self-administered questionnaire, which addressed sociodemographic information, reproductive history, and psychological status.

Infertile women assessed how the suspension of embryo transfer impacted their stress, anxiety, and depressive symptoms using a five-point Likert scale. They were also asked whether they would have proceeded with the transfer during the pandemic. Additional questions explored work/study activity, social isolation practices, confirmed COVID-19 infection, and changes in household income. Emotional symptoms were further assessed using the Depression, Anxiety and Stress Scale - 42 items (DASS-42), a validated psychometric tool comprising 42 questions divided equally into three subscales: depression, anxiety, and stress (Lovibond & Lovibond, 1995). Numerical scores were calculated to quantify symptom severity.

Fertile participants completed a similar questionnaire adapted to their context, focusing on motherhood, partner relationships, and emotional well-being during the pandemic. They also completed the DASS-42.

### Procedures - Phase Two

Six months later, participants from both groups were reassessed using the same DASS-42 scale. Infertile women whose reproductive treatments remained delayed and fertile women were recontacted and invited to complete the follow-up survey. The goal was to compare intra-group (before and after) and inter-group (infertile *vs*. fertile) changes in emotional distress over time.

### Data Analysis Overview

Data from both time points were compiled to analyze emotional symptom trajectories. Intra-group comparisons examined psychological evolution within each group, while inter-group comparisons evaluated differences in stress, anxiety, and depression between groups. The analysis aimed to identify the differential emotional effects of the pandemic based on fertility status.

### Sample Size Calculation

An a priori sample size estimation was conducted was based on anxiety prevalence of approximately 7% (O’Donnell *et al*., 2013, Vahratian *et al*., 2011) in the general population and up to 18% among infertile women (Massarotti *et al*., 2019). Considering a projected 15% increase due to the pandemic (Ćosić *et al*., 2020), a minimum of 32 participants per group was needed for adequate statistical power. To compensate for potential attrition, the sample size was increased by 30%, resulting in a minimum of 42 participants per group.

### Statistical Analysis

Data were organized in Microsoft Excel and analyzed using SPSS version 20.0 (SPSS Inc., Chicago, IL, USA). Normality was assessed using the Shapiro-Wilk test, and homogeneity of variances by Levene’s test. For normally distributed data, comparisons between groups were performed using Student’s t-test or one-way ANOVA. Intra-group comparisons used paired t-tests. For nonparametric data, Wilcoxon or Mann-Whitney U tests were applied. Categorical variables were analyzed using chi-square or Fisher’s exact tests. Logistic regression was used to explore associations between variables. To control for potential confounding by age, an analysis of covariance (ANCOVA) was performed for DASS-42 domains (anxiety, stress, and depression) at both assessment phases, with age included as a covariate. Adjusted mean differences and 95% confidence intervals were reported. Statistical significance was defined as *p*<0.05 (two-tailed).

## RESULTS

The demographic characteristics of the infertile and fertile groups are shown in **Table 1**. The infertile group had a significantly higher mean age compared to the control group (37.8 *vs*. 35.5 years; *p*<0.001). There was no significant difference in body mass index (BMI), weight, or height between groups. As expected, the control group reported a significantly higher number of pregnancies (1.4 *vs*. 0.1; *p*<0.001) and abortions (0.5 *vs*. 0.06; *p*<0.001). Educational level distribution was similar between groups (*p*=0.750), with most participants holding a university or postgraduate degree.

**Table 1:** Demographic characteristics of the infertile and fertile groups

Variable	Infertile Group n=71	Fertile Group n=117	p-value
Age (years)	37.8±4.6	35.5±4.6	<0.001^^*^^
Years of Infertility	3.2±2.3	-	-
Pregnancy (n)	0.1±0.3	1.4±0.6	<0.001^^*^^
Abortion (n)	0.06±0.02	0.5±0.3	<0.001^^*^^
Body Mass Index (kg/m^2^)	24.6±3.2	24.1±2.9	0.670^^*^^
Weight (kg)	65.9±9.3	64.8±9.2	0.432^^*^^
Height (m)	1.61±0.06	1.62±0.06	0.360^^*^^
Educational level - High School - Undergraduate - Graduate - Master's degree - Doctorate	2.8% (2) 21.1% (15) 57.7% (41) 9.9% (7) 8.5% (6)	6.0% (7) 23.9% (28) 54.7% (64) 10.3% (12) 5.1% (6)	0.750^†^

^^*^^ Student’s t-test

† Chi-square test

When the infertile group was asked whether they would proceed with embryo transfer during the pandemic, nearly half of the participants (48.3%) expressed willingness to continue treatment, 22.5% stated they would definitely proceed, and 25.8% would probably do so. On the other hand, 30.3% reported they would probably not proceed, 7.9% said they would not proceed at all, and 13.5% were uncertain.

The occupational activities and isolation behaviors reported by infertile and fertile women during the COVID-19 pandemic are show in Table 2. Regarding occupational activities and study routines during the pandemic, a significant difference was observed between infertile and fertile women (*p*=0.002). While the majority of fertile participants reported exclusively working or studying from home (56.7% *vs*. 38.2%), infertile women more frequently reported a mixed routine of on-site and remote work (39.3% *vs*. 15.3%). Household care was also more commonly reported among fertile participants (20.4% *vs*. 11.2%). These findings indicate distinct occupational and domestic adjustments between the groups, reflecting different vulnerabilities and coping strategies during the COVID-19 pandemic. Regarding isolation behavior (Table 2), no significant differences were found (*p*=0.146).

**Table 2:** Self-assessment of work/study activity and COVID-19-related isolation behavior among infertile and fertile women

Variable	Infertile Group (n=71)	Fertile Group (n=117)	p-value^*^
Self-assessment on work and study activity during COVID-19 Did not change, continued normally Continued exclusively through home office Partially on-site and partially home office Work with household care Dismissed due to pandemic	10.1% (7) 38.2% (27) 39.3% (28) 11.2% (8) 1.1% (1)	6.4% (8) 56.7% (68) 15.3% (18) 20.4% (24) 1.3% (1)	0.002
Social isolation and COVID-19 exposure Did not quarantine Quarantined as precaution Suspicious symptoms without testing Confirmed COVID-19 (RT-PCR or serology) Hospitalization due to COVID-19	15.7% (14) 65.2% (58) 7.9% (7) 9.0% (8) 2.2% (2)	3.8% (6) 78.3% (123) 5.1% (8) 12.7% (20) 0% (0)	0.146
^*^ *p*-values calculated using Fisher’s exact test			

Table 3 shows the self-perceived psychological impact of the COVID-19 pandemic. Infertile women more frequently perceived the postponement of treatment as emotionally detrimental compared to the fertile group (75.8% *vs*. 69.6% reporting “much worse” or “slightly worse” and 21.4% *vs*. 10.7% “slightly better” or “much better”, respectively; *p*=0.017). In relation to anxiety, both groups reported worsening, but puerperal women showed a significantly higher proportion of negative responses (80.9% *vs*. 70.8%; *p*=0.003). Stress-related perceptions followed the same pattern, with fertile women more frequently reporting “much worse” or “slightly worse” (82.8% *vs*. 52.8%; *p*<0.001). Regarding depressive symptoms, fertile participants again reported a greater worsening compared to infertile women (45.2% *vs*. 37.1%; *p*=0.022). These findings indicate that while infertile patients internalized the burden of treatment suspension, puerperal women experienced greater overall psychological vulnerability during the pandemic.

**Table 3:** Self-assessment of psychological impact during the COVID-19 pandemic of the infertile (n=71) and fertile (n=117) groups.

Question	Group	Much worse (%)	Slightly worse (%)	No difference (%)	Slightly better (%)	Much better (%)	p-value^*^
Perceived impact of delaying treatment/motherhood	Infertile Fertile	26.1% 32.6%	49.7% 37.0%	13.4% 9.0%	5.0% 18.0%	5.7% 3.4%	0.017
Anxiety related to treatment/motherhood	Infertile Fertile	37.1% 38.9%	33.7% 42.0%	12.4% 17.2%	11.2% 1.9%	5.6% 0.0%	0.003
Stress related to treatment/motherhood	Infertile Fertile	22.5% 37.0%	30.3% 45.8%	24.7% 15.3%	13.5% 1.3%	9.0% 0.6%	<0.001
Depressive symptoms related to treatment/motherhood	Infertile Fertile	12.4% 17.2%	24.7% 28.0%	43.8% 49.7%	6.7% 3.2%	12.4% 1.9%	0.022
^*^Chi-square or Fisher’s exact test (Monte Carlo simulation), as appropriate.							

The comparison of anxiety, stress, and depression levels between infertile and fertile women, assessed using the DASS-42 scale during the early phase of the COVID-19 pandemic are show in Table 4. During the early phase, infertile women reported significantly higher levels of stress (7.7 *vs*. 5.6, *p*=0.018) and depression (4.0 *vs*. 2.3, *p*=0.002) compared to the fertile group. Anxiety levels were equal between the infertile and fertile groups (3.4 *vs*. 2.7, *p*=0.389). The comparison of the psychological outcomes between the groups six months later the begin off COVID-19 show that stress (3.7 *vs*. 3.1), depression (4.0 *vs*. 3.5), and anxiety (5.8 *vs*. 5.4) levels were similar between groups, with no statistically significant differences observed.

**Table 4:** DASS-42 assessment at the beginning (Phase 1) and after six months (Phase 2) of the COVID-19 pandemic of the infertile (n=71) and fertile (n=117) groups

Initial Assessment	Infertile Group (µ±SD)	Fertile Group (µ±SD)	p-value^*^
Anxiety Stress Depression	3.4±3.13 7.7±4.59 4.0±3.77	2.7±3.14 5.6±4.29 2.3±3.33	0.389 0.018 0.002
**After 6 Months**	**Infertile Group (µ±SD)**	**Fertile Group (µ±SD)**	p-value^*^
Anxiety Stress Depression	5.8±3.66 3.7±2.61 4.0±2.60	5.4±4.26 3.1±2.42 3.5±3.08	0.666 0.201 0.446
^*^Student’s t-test.			

The intraand inter-group comparisons of DASS-42 scores (anxiety, stress, and depression) between the initial phase of the COVID-19 pandemic and six months later are show in Table 5. In the infertile group, there was a significant increase in anxiety from baseline to follow-up (3.4 *vs*. 5.8; *p*<0.001), accompanied by a significant reduction in stress (7.7 *vs*. 3.7; *p*<0.001). Depressive symptoms remained stable over time (4.0 *vs*. 4.0; *p*=0.980). In the fertile group, anxiety also increased significantly (2.7 *vs*. 5.4; *p*<0.001), along with a rise in depressive symptoms (2.3 *vs*. 3.5; *p*=0.018), while stress levels showed a significant decrease (5.6 *vs*. 3.1; *p*<0.001).

**Table 5:** Intraand inter-group comparison of DASS-42 scores during the COVID-19 pandemic of the infertile (n=71) and fertile (n=117) groups

Domain	Group	Baseline (µ±SD)	6 Months (µ±SD)	∆ Change	p-value^*^	p-value†
Anxiety	Infertile Fertile	3.4±3.13 2.7±3.14	5.8±3.66 5.4±4.26	+2.4 +2.7	<0.001 <0.001	0.463
Stress	Infertile Fertile	7.7±4.59 5.6±4.29	3.7±2.61 3.1±2.42	-4.0 -2.5	<0.001 <0.001	0.011
Depression	Infertile Fertile	4.0±3.77 2.3±3.33	4.0±2.60 3.5±3.08	0.0 +1.20	0.980 0.018	0.035

^^*^^Intra-group comparison, baseline and after 6 months: Student’s paired t-test.

† Inter-group comparison, baseline and after 6 months: multivariate analysis between groups.

In the inter-group analysis, the reduction in stress was more pronounced among infertile women (∆ -4.0 *vs*. -2.5; *p*=0.011), whereas the progression of depressive symptoms was greater in the fertile group (∆ +1.2 *vs*. 0.0; *p*=0.035). For anxiety, there was no significant difference in the magnitude of change between groups (∆ +2.4 *vs*. +2.7; *p*=0.463).

After controlling for age, infertile women continued to show significantly higher depression (*p*=0.031) and stress (*p*=0.042) scores at Phase 1, indicating that these differences were not solely explained by age. When analyzing both phases together, age-although significantly different between groups-did not exert a significant influence on the DASS-42 outcomes. These results confirm that the observed psychological differences are primarily related to fertility status rather than age (Table 6).

**Table 6:** ANCOVA adjusted for age in DASS-42 scores among infertile (n=71) and fertile (n=117) women

DASS-42 Domain	Infertile (adj. ‡)	Fertile (adj. ‡)	p (Group)^*^	β (Age)	p (Age)†
Anxiety (Phase 1)	2.79	2.17	0.353	-0.050	0.504
Stress (Phase 1)	6.49	4.89	0.042	-0.046	0.631
Depression (Phase 1)	3.43	2.01	0.031	0.067	0.360
Anxiety (Phase 2)	4.72	4.43	0.730	-0.017	0.857
Stress (Phase 2)	2.88	2.39	0.364	0.049	0.425
Depression (Phase 2)	3.12	2.98	0.827	0.038	0.582
^*^ ANCOVA with age as a covariate. † β represents the estimated coefficient for age in each model. **‡**adj.=adjusted mean, estimated after controlling for age.					

### Power Analysis

After the study was completed, the sample power calculation was performed. A post hoc power analysis was conducted using two-tailed t-tests (α=0.05) to evaluate the statistical power for anxiety, stress, and depression outcomes. The sample size provided sufficient power for detecting moderate effect sizes, with power estimates above 90% for stress and depression comparisons between groups.

## DISCUSSION

This study demonstrates that the COVID-19 pandemic exerted a differential psychological impact on infertile and fertile women. In the early phase of the pandemic, when assisted reproductive treatments were suspended, infertile patients presented significantly higher levels of stress and depression compared to fertile women. These findings are consistent with prior literature showing that infertility itself is associated with substantial psychological burden, which may be exacerbated during treatment interruption or uncertainty (Aimagambetova *et al*., 2020; Maroufizadeh *et al*., 2015). Interestingly, after six months, stress levels decreased in both groups, with a more pronounced reduction observed among infertile women. This suggests a possible adaptation to the lockdown environment or temporary relief from the pressures of fertility treatments (Fancourt *et al*., 2021). However, anxiety levels significantly increased in both groups over time, potentially reflecting prolonged uncertainty, fear of illness, and concern for family well-being (Bu *et al*., 2023).

In contrast to stress and anxiety, depressive symptoms remained stable in infertile women but significantly worsened in the fertile group. These women were in the postpartum period, stage that may increase psychological vulnerability due to hormonal changes, isolation, or childcare burden. Meta-analyses conducted during the COVID-19 pandemic revealed that rates of postpartum depression increased from a pre-pandemic baseline of approximately 25% (Sahebi *et al*., 2024). Furthermore, reduced social support, crucial for postpartum adjustment, was significantly associated with increased depression, anxiety, and impaired maternal-infant bonding during the pandemic (White *et al*., 2023). These findings underscore that different mental health domains (anxiety, stress, depression) may evolve independently and respond differently to external stressors.

The longitudinal design of our study, with repeated DASS-42 assessments, allowed the tracking of emotional trajectories over time. While initial differences favored the fertile group, follow-up results revealed a convergence in stress and anxiety levels, and even an inversion in depressive symptoms. These results emphasize that emotional health is dynamic and context-dependent, and that psychological screening should be routinely incorporated into fertility care.

Beyond statistical significance, the clinical meaning of the DASS-42 score changes should also be considered. In the infertile group, the mean anxiety score increased from 3.4 to 5.8, representing a transition from the normal to the mild anxiety range according to validated DASS-42 cut-offs. A similar trend was observed among fertile women, whose anxiety rose from 2.7 to 5.4, also shifting into the mild category. These findings indicate that both groups experienced clinically perceptible increases in anxiety during the pandemic, reflecting heightened uncertainty, fear of infection, and social isolation. Conversely, stress levels decreased significantly in both groups, moving from moderate to normal ranges, suggesting emotional adaptation and coping over time. However, depressive symptoms evolved differently: while infertile women maintained stable scores (remaining within the normal range), fertile women exhibited a mild but significant increase, shifting from normal to mild depression, consistent with reports of postpartum vulnerability during prolonged lockdowns (Caffieri *et al*., 2024; Safi-Keykaleh *et al*., 2022). Collectively, these changes underscore that even mild symptom fluctuations may hold clinical importance, emphasizing the need for ongoing psychological monitoring and individualized support in both reproductive contexts (Bagade *et al*., 2022; Hajihasani & Ekhtiari Amiri, 2023).

In addition to the DASS-42 scale, we also administered self-report questions to patients, and these questions offered valuable subjective insights into how patients perceived their emotional state during the pandemic. Among infertile women, nearly 70% reported that postponing fertility treatment made them feel “much worse” or “somewhat worse” in their current life context and over 48% expressed willingness to proceed with embryo transfer despite pandemic risks. These findings align with international reports showing that treatment suspension led to frustration, loss of control, and emotional distress among patients (Gordon & Balsom, 2020). These perceptions echoed across emotional domains: over 70% rated the postponement as having worsened their anxiety, and over 50% reported a negative impact on their stress and depressive symptoms.

Fertile women also expressed substantial emotional distress during the pandemic due to the need to care for their children, with over 80% reporting increased anxiety and stress. Interestingly, while some of these self-perceived impacts aligned with the DASS-42 findings, others revealed a broader emotional burden not fully captured by standardized scales. This discrepancy highlights the value of combining validated instruments with open-ended or self-report tools to gain a more detailed understanding of patients’ psychological experiences (Wedner-Ross *et al*., 2022).

Our self-assessment results revealed distinct perceptions between groups. Infertile women more frequently perceived the postponement of treatment as emotionally detrimental, reporting greater worsening in their well-being due to delays. In contrast, puerperal women-although not directly affected by treatment suspension-perceived higher levels of anxiety, stress, and depressive symptoms in their daily lives. This indicates that while infertile patients mainly internalized the burden of delayed reproductive planning, puerperal women reported a heavier overall psychological toll associated with the postpartum period during the pandemic. These findings align with studies demonstrating that infertility amplifies treatment-related distress (Kiani *et al*., 2021), while postpartum women remain vulnerable to anxiety and depressive symptoms intensified by external crises (Galletta *et al*., 2022). Together, these results underscore the importance of tailored psychological support, considering both reproductive context and self-perceived emotional burden.

Although age was significantly higher in the infertile group, an analysis of covariance (ANCOVA) controlling for age confirmed that this variable did not significantly influence DASS-42 anxiety, stress, or depression scores at either phase. These findings suggest that the greater emotional burden observed among infertile women was not explained by age differences, but rather by factors inherent to infertility and treatment interruption. Nevertheless, age should be acknowledged as a potential confounder, as prior studies report mixed evidence-some showing associations between higher age and greater distress in infertile women (Lakatos *et al*., 2017), while others find no meaningful age effect on distress measures (Chi *et al*., 2016).

Although this study presents some limitations, such as the use of a sample from a single fertility center, which may limit the generalizability of the findings, it also has several strengths. The inclusion of both infertile and fertile women allowed for direct comparison between two distinct reproductive realities. Furthermore, the longitudinal design, with two DASS-42 assessments at different pandemic phases, is relatively uncommon in psychological research conducted during public health crises.

Despite the significant age difference between groups, an ANCOVA controlling for age demonstrated that this variable did not influence anxiety, stress, or depression outcomes. Nonetheless, age should still be acknowledged as a potential confounder, and future studies with age-matched cohorts are warranted. Other unmeasured factors, such as prior psychiatric history, socioeconomic status beyond educational level, or pre-existing stressors, could also have influenced emotional responses. Additionally, the findings were derived from a specific pandemic context, which may limit their applicability to non-crisis situations.

A key strength of this study lies in its adequate statistical power. Post hoc analysis confirmed over 90% power to detect moderate differences in stress and depression between groups, reinforcing the reliability of the results. Overall, these findings contribute valuable insights into how women with different reproductive statuses respond emotionally over time during major societal disruptions and underscore the importance of incorporating psychological screening and tailored mental health support into fertility care.

## CONCLUSION

The suspension of infertility treatments during the COVID-19 pandemic had a significant psychological impact on women undergoing assisted reproduction. This study demonstrates that the COVID-19 pandemic had a differentiated psychological impact on infertile and fertile women, with distinct trajectories across all emotional domains. At the onset of the pandemic, infertile patients experienced significantly higher levels of stress and depressive symptoms compared to fertile women, reflecting the emotional burden of treatment delays and uncertainty. Over time, however, stress decreased more markedly among infertile women, while fertile women experienced a significant increase in depressive symptoms. Anxiety levels increased in both groups, highlighting the widespread and lasting influence of the pandemic on mental health.

These results emphasize that psychological responses to crises are not uniform, but evolve according to reproductive context and individual vulnerability. Infertile women initially suffered more from treatment suspension, while postpartum women reported greater anxiety and depression during follow-up. Taken together, these findings reinforce the importance of integrating systematic and individualized psychological support into fertility care, both in times of public health emergencies and in routine practice, to address patients’ specific needs and improve emotional resilience throughout the reproductive journey.
